# Rehabilitation Process Issues and Functional Performance after Total Hip and Knee Replacement

**DOI:** 10.3390/healthcare9091126

**Published:** 2021-08-30

**Authors:** Alexander S. Fedonnikov, Elena A. Andriyanova, Anton R. Kiselev, Igor A. Norkin

**Affiliations:** 1 Institute of Public Health, Health Management and Humanitarian Problems of Medicine, Research Institute of Traumatology, Orthopedics and Neurosurgery, Saratov State Medical University, 410012 Saratov, Russia; fedonnikov@mail.ru (A.S.F.); andriyanova@yandex.ru (E.A.A.); norkin@sarniito.com (I.A.N.); 2 National Medical Research Center for Therapy and Preventive Medicine, 101990 Moscow, Russia

**Keywords:** total hip replacement, total knee replacement, rehabilitation management, social survey

## Abstract

(1) Background: to ensure satisfactory outcomes in patients who have undergone total hip or knee replacement, it is crucial to prioritize postoperative rehabilitation process and its management rather than the successful surgery alone. The goal of our study was to investigate the outcomes of rehabilitation process after the total hip or knee replacement, including local orthopedic follow-up, communication with local health authorities, patients’ satisfaction regarding rehabilitation, and their functional performance after the surgery. (2) Methods: the study included 523 patients who underwent total hip replacement, and 650 patients who underwent total knee replacement. All patients were surveyed via formalized phone interviews containing questions related to postoperative rehabilitation parameters and outcomes. (3) Results: in postsurgical period, nearly 70% of patients had regular local orthopedic follow-up. Lack of the latter at the local level was indicated by approximately 10% of patients, and the rest of the respondents note the practice of sporadic follow-up. Half of patients reported pains of various severity. Good physical activity (e.g., ability to walk unassisted within their residential district) was acknowledged by about a quarter of patients. Ability to walk unassisted was reported by just 54.5% of patients. (4) Conclusions: the disproportion between generally high patient satisfaction of rehabilitation management (>80%) and low feedback level on the part of local health authorities (9.4%) demonstrated lack of communication between the key parties involved in the rehabilitation process.

## 1. Introduction

Osteoarthritis of knee and hip joints is among the most significant disorders of the musculoskeletal system and connective tissue. It has high prevalence rate due to progressive population aging, diminishing physical activity, along with growing obesity and injury rates [1,2]. Considerable impact of knee and hip osteoarthritis on work capability and quality of life represents the social aspect of this problem [3]. Principal surgical approach to treating severe hip and knee osteoarthritis is total joint replacement [4,5,6]. For example, the prevalence rates of total hip replacement (THR) and total knee replacement (TKR) in the USA population in 2010 were 0.83% and 1.52%, respectively [7]. Population analysis of patients who underwent THR or TKR is habitually performed using specialized registries [8,9,10]. In Russia, there is no unique national registry for such patients, and, consequently, there are no apparent epidemiological data [11]. Moreover, in the last decade, persistent growth of THR and TKR surgeries was observed in Russia [12]. To ensure satisfactory outcomes in patients who underwent THR or TKR, it is vital to prioritize their postoperative rehabilitation process and its management rather than the successful surgery alone [13]. Sufficiently large cohorts of concerned postoperative patients are formed, which requires to investigate possible differences in rehabilitation evaluation by THR vs. TKR patients in order to provide the most effective individual approach to their rehabilitation process. The situation is complicated by the personnel shortage in outpatient facilities for orthopedic services. It is worth noting that the shortage rate, according to the Ministry of Healthcare of Russia is about 40% [14,15].

Hence, the described situation determines the goal of this study which was to investigate the outcomes of rehabilitation process after the total hip or knee replacement, including local orthopedic follow-up, communication with local health authorities, patients’ satisfaction regarding rehabilitation, and their functional performance after the surgery. 

## 2. Materials and Methods

### 2.1. Study Design

Our study involved 1173 patients who met the following enrollment criteria:

(i) They underwent THR or TKR surgery between 1 January 2015 and 30 June 2016, at Research Institute of Traumatology, Orthopedics and Neurosurgery of Saratov State Medical University;
(ii) They were diagnosed with unilateral coxarthrosis (M16) or gonarthrosis (M17);
(iii) They were over 18 years of age.

The exclusion criteria for prospective study participants were as follows:

(i) Their contact information in medical records was absent or outdated;
(ii) They could not be reached by phone calls;
(iii) They did not respond to all questions of the survey.

From the local database of medical records, we obtained the following data for each patient included in our study: age, gender, place of residence, ICD-10 code of their diagnosis, and surgery type. Bilateral osteoarthritis was excluded in order to investigate the rehabilitation process in patients who first encountered the osteoarthritis problem.

All patients were surveyed in order to collect data regarding their rehabilitation and its outcomes.

### 2.2. Participants

Patients included in the study were distributed among two groups. The first group was composed of subjects (*n* = 523; 65.2% females) who underwent THR. The second group comprised the patients (*n* = 650; 88.2% females) who underwent TKR. Such patient assignment to the study groups was performed in order to explore possible differences in rehabilitation evaluation by THR vs. TKR patients.

Subjects participating in our study reside permanently in 24 regions of 5 federal districts in Russia. Respondents from Southern (39.3%) and North Caucasian districts (31.7%) prevailed.

This study was approved by the Ethics Committee (Minutes No. 1 of 5 September 2016) at Saratov State Medical University (Saratov, Russia). The written informed consent was obtained from all participants.

### 2.3. Questionnaire Survey

All patients were surveyed via formalized phone interviews. Each survey session lasted approximately 15 ± 2 min (M ± SD) and was recorded. Prior to the survey, the interviewers were thoroughly instructed about the study goal, the structure of the questionnaire, and the manner of conversing with respondents. The full text of the questionnaire is presented in Appendix A. The questionnaire was developed at Research Institute of Traumatology, Orthopedics and Neurosurgery of Saratov State Medical University. It includes the questions about the most important issues of rehabilitation process and its functional outcomes.

Our survey contained the questions related to evaluation of postsurgical rehabilitation process parameters (regularity of a local orthopedic monitoring, communication with local health authorities, evaluation of local rehabilitation management), and rehabilitation outcomes (evaluation of chronic pain, physical activity, limb function and anatomical changes). 

For self-rated evaluation of the chronic pain severity by the patients, we used the principle of verbal descriptor scale (VDS). To fill in Section 2.1 of the questionnaire, the following VDS categories were used to explain the patients how to describe the pain severity: No pain (self-explanatory), Mild (i.e., annoying pain), Nagging (i.e., uncomfortable pain), Distressing (i.e., miserable pain), Intense (i.e., horrible pain), Worst possible (i.e., unbearable pain). 

Only entirely (100%) filled questionnaires were included in further statistical analysis.

### 2.4. Statistical Analysis

We used the Shapiro–Wilk test to examine whether the distribution of a variable is normal. We reported median, and lower and upper quartiles for the variables that were not normally distributed and mean and standard deviation for the normally distributed variables. Binary variables were presented as proportions (in percentages) with 95% confidence intervals. We applied the Chi-squared (χ^2^) test to compare the proportions among the groups. 

## 3. Results

**Figure 1:**
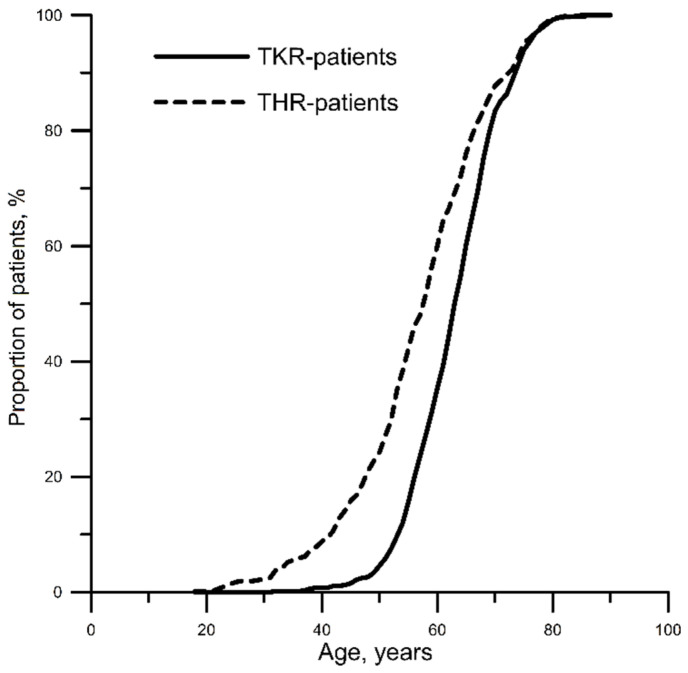
Patient age distribution versus surgery type. THR, total hip replacement; TKR, total knee replacement.

The study encompassed 1173 patients (77.9% females) with an average age of 61 (55, 68) years, who underwent THR or TKR. After distributing the patients among two study groups, THR patients (*n* = 523) were, on average, 54 (39, 70) years of age, whereas TKR patients (*n* = 650) were, on average, 59 (45, 72) years old. Age distribution in groups of patients is presented in Figure 1. The latter reveals the following age trends vs. surgery type: THR prevailed in subjects 18–52 years old, while those 53–82 years old had primarily TKR.

Average (mean ± standard deviation) length of stay at inpatient facility was 8.8 ± 0.3 days.

As for time that has elapsed after the surgery, absolute majority of subjects passed the early postsurgical period (95.8% of THR-patients and 94.4% of TKR-patients) (Table 1). Thus, the distribution of patients allows the regularity of outpatient visits to healthcare professionals to be correctly estimated. Bearing in mind the absence of statistically significant differences in postsurgical time for THR vs. TKR patients, and the striking predominance of patients with postoperative period of 6 months or more, further data analysis regarding rehabilitation and outcomes was carried out without considering the postsurgical time frame all.

In postsurgical period, about 70% of patients had regular local orthopedic follow-up, while 10% had none at all. We established statistically significant difference of orthopedic follow-up between patients who underwent THR vs. those who underwent TKR: 1.5 more patients who underwent TKR do not have local orthopedic postsurgical monitoring. About 70% of patients in both groups confirmed that they visited a local orthopedic surgeon voluntarily. However, for THR patients, it was more distinctive (74.2% against 68.7%) at a statistically significant level (Table 2). 

Overall, no more than 9% of patients in both groups indicated active rehabilitation support on the part of local health authorities; about 10% of subjects in both groups specified actual absence of rehabilitation at the local level. Voluntary consultation with the orthopedic surgeon who has performed the surgery was admitted by 7.6–10.6% of patients in both groups (Table 2).

Evaluation results demonstrated that about 70% of patients were fully satisfied with the local management of their postsurgical rehabilitation. Approximately 20% of surveyed subjects were unequivocally dissatisfied. Major causes for dissatisfaction were associated with a poor healthcare quality (no result after visiting a local orthopedic surgeon) and absence of a local orthopedic surgeon. No statistically significant differences between the groups were revealed (Table 3).

The analysis of chronic pain in patients showed that nearly half of them experienced pain of some degree of severity. Absence of pain was more often stated by THR patients; whereas nagging, uncomfortable pain was more frequently admitted by TKR patients at a statistically significant level (Table 4).

Capability to walk independently was stated by slightly over a half of patients. About 43% of patients were able to walk only with an extra support. Fewer than 25% of patients could walk unassisted within their residential district. We established that there were more people capable of moving solely around the house among TKR patients vs. THR patients at a statistically significant level (Table 4).

In fact, three quarters of patients had no difference in limb lengths. TKR patients kept the limb length more often than the subjects of another group, and the difference was statistically significant. Over 90% of patients stated no limb deformations. These findings imply that the surgical stage of treatment was performed correctly. However, nearly 30% of patients had joint excursion (motion range) restrictions (Table 4).

## 4. Discussion

In recent years, we have observed the intensive ongoing development of government programs on hip and knee replacement in Russia [16]. However, the availability of postsurgical rehabilitation to patients is limited due to insufficient resources [17], which could be the cause of the chronic pain persistence and low physical activity of patients. According to other medical and sociological studies, low rehabilitation effectiveness after the hip and knee replacement surgeries is associated with the fact that functional performance evaluation is virtually lacking, as well as with insufficient participation in supporting patients and an incomplete rehabilitation cycle at the outpatient follow-up treatment level in 26% of patients [18].

Our results confirmed our assumption that studying patient opinion about the rehabilitation outcomes regardless of their evaluation of some key points of their physical performance (chronic pain, orthopedic parameters and physical activity) does not benefit the correct analysis of rehabilitation management effectiveness. According to our data, over 80% of patients expressed their satisfaction with rehabilitation quality, albeit the presence of permanent pain was attributed to nearly 45% of surveyed subjects.

The above assumption and obtained results were confirmed in different studies of joint replacement outcomes and rehabilitation strategies. According to some published research projects conducted in other countries, the main complaints of patients in postsurgical period were related to their lameness and pain. These issues may bother patients for several years after the surgery, even in case of implant stability confirmed by X-ray examination [19]. An importance of monitoring the chronic pain as a major factor, influencing quality of life after the surgery, was described in other publications [20,21].

Subjective evaluation of a patient’s physical activity should be also taken into consideration in terms of the walking distance and the motion range of both operated and unoperated hip joints [22].

Difference in leg lengths is often considered a problem after the THR surgery, and may adversely affect an otherwise favorable outcome. Furthermore, it has been associated with a patient dissatisfaction, and remains one of the most common reasons for litigation against the orthopedic community [23].

The clinical implication of the obtained results is associated with establishing the continuous feedback programs between patients and doctors (orthopedists, rehabilitation therapists, etc.) at the level of medical institutions, which would effectively monitor and control long-term medical and social results of complicated surgeries such as THR/TKR.

In order to improve this routine in the future, it is necessary to develop and introduce the feedback social services using digital technology of interactive online communication (web-platforms supported by medical institutions serving THR/TKR providers), which could be an effective organizational tool for controlling the long-term medical and social consequences of THR/TKR surgeries [24,25].

Over last few years, several studies dealing with chronic pain, patient mobility, necessity and advantages of a long-term rehabilitation for THR and TKR patients, including social surveys, were published [26,27]. We can also find very good examples of effective management strategies [28,29]. Hence, the results of the present study correspond to A. Donabedian’s quality assurance methodology with respect to the outcomes [30] and may be potentially useful for researchers, health managers and physicians in the development of patient feedback technologies.

### Limitations

Chronic pain is among the major issues after the joint replacement: e.g., as many as 5–10% of TKR patients experience residual pains [31]. The complexity of the chronic pain assessment should be particularly emphasized: different approaches to pain intensity description have been published [32]; verbal descriptor scale, along with a numeric rating scale for pain intensity, could be used in medical practice, depending on the preference of a researcher and a respondent [33]. Pain evaluation simplicity in our study allowed obtaining relevant results, while more specific methods should be used for pain assessment with a higher reliability and validity [34,35]. The simplest scale used in our study could cause, without any doubt, some limitations, but still, it has yielded the results meeting the declared goal and methods.

The study participants were recruited from the single center (Research Institute of Traumatology, Orthopedics and Neurosurgery of Saratov State Medical University). They represented approximately 5% of THR/TKR annually performed in Russia [16,17].

The study was conducted in actual clinical practice environment: hence, according to ethical reasons, it involved no patients without rehabilitation.

Among the limitations of our study, we should mention that we did not analyze the dependence of survey results on postsurgical period duration among THR vs. TKR patients.

We analyzed solely general and essential characteristics of THR and TKR patients regardless of the follow-up period duration.

For our data, we consider inappropriate to perform a more detailed analysis, taking into account that most patients were interviewed within 6 months or more after the surgery and that overall distributions of postsurgical time frames in THR vs. TKR patients were statistically comparable (Table 1).

## 5. Conclusions

The study revealed that approximately a quarter of patients who underwent total hip or knee replacement, had no regular local postoperative orthopedic follow-up. The significant disproportion between the high rate of declared generally positive attitude towards the rehabilitation management (over 80% of patients) and low feedback level on the part of local health authorities (9.4%) has demonstrated lack of communication among the key parties in the rehabilitation process. Considering that more than 71% of patients affirmed entire satisfaction with rehabilitation process at the place of their residence, whereas just 54.5% of patients had a capability to walk unassisted, it could be concluded that this finding represented an actual medical and social problem requiring further studying.

## Figures and Tables

**Table 1:** Distribution of patients who underwent THR or TKR according to postsurgical time frame.

Time Elapsed after the Surgery, Months	THR-Patients (*n* = 523)	TKR-Patients (*n* = 650)	*p*-Value
Under 3	4.2 (2.7–6.3)	5.6 (4.0–7.7)	0.27
3–6	8.0 (5.8–10.7)	8.9 (6.8–11.4)	0.58
6–12	25.1 (21.4–29.1)	28.0 (24.6–31.6)	0.27
Over 12	62.7 (58.4–66.9)	57.5 (53.6–61.3)	0.07

Data are presented as proportions (in percentages) with 95% confidence interval. THR, total hip replacement; TKR, total knee replacement.

**Table 2:** Rehabilitation process after the THR or TKR surgery.

No.	Survey Results	THR Patients (*n* = 523)	TKR Patients (*n* = 650)	*p*-Value
Local orthopedic postsurgical monitoring				
1.1.1.	Regular (after 3, 6, 12 months; annually afterwards *) visits to a local orthopedic surgeon	73.2 (69.2–77.0)	66.5 (62.7–70.1)	0.01
1.1.2.	Irregular visits to a local orthopedic surgeon	17.4 ( 14.3–20.9)	19.4 (16.4–22.7)	0.38
1.1.3.	No local orthopedic monitoring	9.2 (6.9–12.0)	13.6 (11.1–16.5)	0.02
1.1.4.	Other	0.2 (0.01–1.1)	0.5 (0.1–1.4)	0.40
Communication with local health authorities				
1.2.1.	Feedback from regional health services representative offering a rehabilitation course	8.6 (6.3–11.3)	8.8 (6.7–11.3)	0.90
1.2.2.	Voluntary visits to a local orthopedic surgeon	74.2 (70.2–77.9)	68.7 (65.0–72.3)	0.04
1.2.3.	Voluntary consultation with the orthopedic surgeon who performed the surgery	7.6 (5.5–10.2)	10.6 (8.3–13.2)	0.08
1.2.4.	Absence of both communication with healthcare system representative and actual rehabilitation	9.4 (7.0–12.2)	11.7 (9.3–14.4)	0.21
1.2.5.	Other	0.2 (0.01–1.1)	0.2 (0.01–0.9)	1.00
Evaluation of postsurgical rehabilitation process at the place of residence				
1.3.1.	Entirely satisfied	71.5 (67.4–75.3)	71.8 (68.2–75.2)	0.91
1.3.2.	Overall, the evaluation of rehabilitation process is positive; however, it needs improvement	8.2 (6.0–10.9)	8.6 (6.6–11.0)	0.81
1.3.3.	Absolutely dissatisfied	19.1 (15.8–22.7)	18.6 (15.7–21.8)	0.83
1.3.4.	Evaluation is impossible (choosing 1.2.4)	1.1 (0.4–2.4)	0.9 (0.3–2.0)	0.73

Data are presented as proportions (%) with 95% confidence intervals. THR, total hip replacement; TKR, total knee replacement.

**Table 3:** Causes of rehabilitation process dissatisfaction.

No.	Survey Results	THR Patients (*n* = 78)	TKR Patients (*n* = 99)	*p*-Value
1.3.3.1.	Absence of a local orthopedic surgeon	21.8 (13.2–32.6)	30.3 (21.5–40.4)	0.21
1.3.3.2.	Lack of time for visiting a local orthopedic surgeon	10.3 (4.6–19.3)	10.1 (5.0–17.8)	0.97
1.3.3.3.	Poor healthcare quality (no result after visiting a local orthopedic surgeon)	26.9 (17.5–38.1)	30.3 (21.5–40.4)	0.62
1.3.3.4.	Other	41.0 (30.0–52.7)	29.3 (20.6–39.3)	0.11

Data are presented as proportions (%) with 95% confidence intervals. THR, total hip replacement; TKR, total knee replacement. Note that the total number of patients does not match the same number in question 1.3.3 (see Table 2), because not all respondents indicated the reasons of rehabilitation management dissatisfaction.

**Table 4:** Rehabilitation outcomes/Functional performance.

No.	Survey Results	THR Patients (*n* = 523)	TKR Patients (*n* = 650)	*p*-Value
Chronic pain evaluation				
2.1.1.1.	No pain	56.4 (52.0–60.7)	47.0 (43.1–50.9)	<0.01
2.1.1.2.	Mild, annoying pain	25.1 (21.4–29.1)	26.2 ( 22.9–29.8)	0.67
2.1.1.3.	Nagging, uncomfortable pain	14.2 (11.3–17.5)	20.7 (17.7–24.0)	<0.01
2.1.1.4.	Distressing, miserable pain	3.1 (1.8–5.0)	4.0 ( 2.6–5.8)	0.41
2.1.1.5.	Intense, horrible pain	0.8 (0.2–2.0)	1.5 (0.7–2.8)	0.27
2.1.1.6.	Worst possible, unbearable pain	0.4 (0.1–1.4)	0.6 (0.2–1.6)	0.63
Evaluation of physical activity, limb function and anatomic changes				
*Ability to walk*				
2.2.1.1.	Unable to walk	2.1 (1.1–3.7)	3.8 (2.5–5.6)	0.09
2.2.1.2.	Able to walk with extra support (cane, crutches)	40.5 (36.3–44.9)	44.0 (40.1–47.9)	0.23
2.2.1.3.	Able to walk independently	57.4 (53.0–61.7)	52.2 (48.3–56.1)	0.08
*Range of walking without rest*				
2.2.2.1.	Able to move inside the house	14.5 (11.6–17.8)	19.8 (16.8–23.1)	0.02
2.2.2.2.	Able to reach nearby facilities	29.6 (25.7–33.7)	31.5 (27.9–35.2)	0.48
2.2.2.3.	Able to walk several blocks	31.2 (27.3–35.4)	27.5 (24.1–31.1)	0.17
2.2.2.4.	Able to walk unassisted within the residential district	24.7 (21.1–28.6)	21.2 (18.1–24.6)	0.16
*Anatomical and functional changes of the limb*				
2.2.3.1.	Joint excursion (motion range) is restricted	26.0 (22.3–30.0)	31.1 (27.6–34.8)	0.06
2.3.1.1.	No difference in limb lengths	72.9 (68.9–76.7)	78.2 (74.8–81.3)	0.04
2.3.2.1.	No limb deformations	93.9 (91.5–95.8)	91.7 (89.3–93.7)	0.15

Data are presented as proportions (%) with 95% confidence intervals. THR, total hip replacement; TKR, total knee replacement.

## Data Availability

Not applicable.

## Appendix A

SURVEY

**“Monitoring of Rehabilitation Process after Total Hip and Knee Replacement”**

After self-introduction, an interviewer informs a surveyed person about the goal of the study and kindly asks them to accurately answer the following questions. While working with the questionnaire, an interviewer should circle the selected items or write down the answers.

1. Rehabilitation process after THR/TKR surgery.

1.1. Please, describe your local orthopedic follow-up after the surgery?

1.1.1. Regular (after 3, 6, 12 months; annually afterwards *) visits to a local orthopedic surgeon

1.1.2. Irregular visits to a local orthopedic surgeon

1.1.3. No local orthopedic follow-up

1.1.4. Other (indicate) __________________________________________

1.2. How your postoperative communication with local health authorities was organized?

1.2.1. Feedback from regional health services representative offering a rehabilitation course

1.2.2. Voluntary visits to a local orthopedic surgeon

1.2.3. Voluntary consultation with the orthopedic surgeon who has performed the surgery

1.2.4. Absence of communication with a healthcare system professional and actual rehabilitation

1.2.5. Other (indicate) ______________________________________

1.3. Please, evaluate postsurgical rehabilitation process at your place of residence.

1.3.1. Entirely satisfied

1.3.2. Overall, the evaluation of rehabilitation process is positive; however, it needs improvement

1.3.3. Absolutely dissatisfied.

Please, indicate causes of rehabilitation process dissatisfaction (relating to question 1.3.3):

1.3.3.1. Absence of a local orthopedic surgeon

1.3.3.2. Lack of time for visiting a local orthopedic surgeon

1.3.3.3. Poor healthcare quality (no result after visiting a local orthopedic surgeon)

1.3.3.4. Other (indicate)___________________________________

1.3.4. Evaluation is impossible (choosing 1.2.4)

2. Rehabilitation outcomes/Functional performance

2.1. Chronic pain evaluation.

2.1.1. Please, describe pain severity according to the simple descriptive scale:

2.1.1.1. No pain

2.1.1.2. Mild, annoying pain

2.1.1.3. Nagging, uncomfortable pain

2.1.1.4. Distressing, miserable pain

2.1.1.5. Intense, horrible pain

2.1.1.6. Worst possible, unbearable pain

2.2. Physical activity, limb function

2.2.1. How can you describe your ability to walk?

2.2.1.1. Unable to walk

2.2.1.2. Able to walk with an extra support (cane, crutches)

2.2.1.3. Able to walk unassisted

2.2.2. What is your range of walking without rest?

2.2.2.1. Able to move around the house

2.2.2.2. Able to reach nearby facilities

2.2.2.3. Able to walk several blocks

2.2.2.4. Walks unassisted within the residential district

2.2.3. Please, evaluate your joint excursion (motion range)

2.2.3.1. Restricted

2.2.3.2. Not restricted

2.3. Anatomical changes of a limb

2.3.1. Is the operated limb length the same as the other one?

2.3.1.1. Yes

2.3.1.2. No

2.3.2. Do you have limb deformations?

2.3.2.1. No deformations

2.3.2.2. Deformations exist

After the survey is completed, an interviewer expresses gratitude for participation in the study.

* An interviewer assesses the regularity of visits for each case, i.e., the time that has elapsed since surgery and up to the interview date, according to the national standards of outpatient care for THR/TKR patients.
